# Diversity of the hepatitis C virus NS5B gene during HIV co-infection

**DOI:** 10.1371/journal.pone.0237162

**Published:** 2020-08-04

**Authors:** Tshegofatso Ngwaga, Ling Kong, Derrick Lin, Cassandra Schoborg, Lynn E. Taylor, Kenneth H. Mayer, Robert S. Klein, David D. Celentano, Jack D. Sobel, Denise J. Jamieson, Caroline C. King, John E. Tavis, Jason T. Blackard

**Affiliations:** 1 Division of Digestive Diseases, University of Cincinnati College of Medicine, Cincinnati, Ohio, United States of America; 2 Miriam Hospital and Department of Medicine, Brown University, Providence, Rhode Island, United States of America; 3 Beth Israel Deaconess Medical Center and the Fenway Institute, Boston, Massachusetts, United States of America; 4 Hudson Infectious Diseases Associates, Briarcliff Manor, New York, United States of America; 5 Department of Epidemiology, Johns Hopkins Bloomberg School of Public Health, Baltimore, Maryland, United States of America; 6 Division of Infectious Diseases, School of Medicine, Wayne State University, Detroit, Michigan, United States of America; 7 Division of Reproductive Health, Centers for Disease Control and Prevention, Atlanta, Georgia, United States of America; 8 Department of Gynecology and Obstetrics, Emory University School of Medicine, Atlanta, Georgia, United States of America; 9 Department of Molecular Microbiology and Immunology, Saint Louis University School of Medicine, St. Louis, Missouri, United States of America; Consejo Superior de Investigaciones Cientificas, SPAIN

## Abstract

Viral diversity is an important feature of hepatitis C virus (HCV) infection and an important predictor of disease progression and treatment response. HIV/HCV co-infection is associated with enhanced HCV replication, increased fibrosis, and the development of liver disease. HIV also increases quasispecies diversity of HCV structural genes, although limited data are available regarding the impact of HIV on non-structural genes of HCV, particularly in the absence of direct-acting therapies. The genetic diversity and presence of drug resistance mutations within the RNA-dependent RNA polymerase (NS5B) gene were examined in 3 groups of women with HCV genotype 1a infection, including those with HCV mono-infection, antiretroviral (ART)-naïve women with HIV/HCV co-infection and CD4 cell count <350 cells/mm^3^, and ART-naïve women with HIV/HCV co-infection and CD4 cell count ≥350 cells/mm^3^. None had ever been treated for HCV infection. There was evidence of significant diversity across the entire NS5B gene in all women. There were several nucleotides and amino acids with distinct distributions across the three study groups, although no obvious clustering of NS5B sequences was observed based on HIV co-infection or CD4 cell count. Polymorphisms at amino acid positions associated with resistance to dasabuvir and sofosbuvir were limited, although the Q309R variant associated with ribavirin resistance was present in 12 individuals with HCV mono-infection, 8 HIV/HCV co-infected individuals with CD4 <350 cells/mm^3^, and 12 HIV/HCV co-infected individuals with CD4 ≥350 cells/mm^3^. Previously reported fitness altering mutations were rare. CD8^+^ T cell responses against the human leukocyte antigen (HLA) B57-restricted epitopes NS5B_2629-2637_ and NS5B_2936-2944_ are critical for HCV control and were completely conserved in 44 (51.8%) and 70 (82.4%) study participants. These data demonstrate extensive variation across the NS5B gene. Genotypic variation may have a profound impact on HCV replication and pathogenesis and deserves careful evaluation.

## Introduction

Globally, an estimated 71 million people have chronic hepatitis C virus (HCV) infection [1]. HCV infection is a major cause of chronic liver disease, hepatocellular carcinoma (HCC), and liver transplantation in the US. There is no vaccine to prevent HCV infection. While significant advances have been made in the treatment of HCV infection in recent years, direct-acting antivirals are costly in some locations and are not available to many individuals.

Genetic diversity is a key feature of HCV. The presence of distinct yet related viral variants within a single infected individual–referred to as *quasispecies* diversity–can impact diagnosis, cell tropism, immunologic escape, viral fitness and pathogenesis, and/or the development of drug resistance [2]. The HCV NS5B protein is an RNA-dependent RNA polymerase that lacks a proofreading mechanism. At the population level, HCV consists of multiple genotypes and subtypes. HCV genotype is a determinant of treatment response, while differences in disease pathogenesis among genotypes may also exist [3–6]. HCV quasispecies can impact transplantation outcome, disease progression, and chronicity [7–20].

NS5B is responsible for the synthesis of negative-sense RNA and subsequently of positive-sense RNA that is incorporated into progeny virions [21, 22]. This essential role in viral replication highlights NS5B –and other non-structural proteins–as major antiviral drug targets. Importantly, the selective pressures that shape non-structural regions of the viral genome are distinct from those targeting structural genomic regions. For instance, highly conserved secondary RNA structures limit NS5B diversity, while immune selection pressures contribute to NS5B variability [23–27]. Immune- or drug-selected mutations in NS5B dramatically reduce viral replication *in vivo*, although compensatory mutations may develop [28, 29]. In a multinational study, variation in consensus viral sequences at known NS3 or NS5B resistance sites was observed in 21.5% of patients, while in another study, consensus NS5B mutations were present in 2.8% of genotype 1a treatment-naïve patients [30, 31]. NS5B variability also impacts pathogenesis as a higher mutation rate is associated with elevated ALT levels, and NS5B enzymatic activity positively correlates with ALT levels [32, 33].

Epidemiologic studies clearly indicate that HIV/HCV co-infection is associated with enhanced HCV replication, increased fibrosis, and the development of liver disease. HIV also increases quasispecies diversity of HCV structural genes. In contrast, HIV results in lower HCV-specific immune responses that may reduce selective pressures targeting immune epitopes. Despite the growing importance of NS5B as an emerging target of HCV infection, limited data are available regarding its genotypic variability and phenotypic properties in the absence of directly acting therapies [34–38]. We examined the genetic diversity and presence of drug resistance mutations within the NS5B gene of treatment naïve HCV monoinfected and HCV/HIV co-infected women using next generation sequencing technology.

## Methods

### Study population

The HIV Epidemiologic Research Study (HERS) was established in 1993 to define the biological, psychological, and social effects of HIV infection in US women [39]. Study procedures and sample collection were approved by the Institutional Review Boards (IRB) at each of the four institutions–Johns Hopkins University School of Hygiene and Public Health, Baltimore, MD; Montefiore Medical Center, Bronx, NY; Brown University, Providence, RI; and Wayne State University School of Medicine, Detroit, MI. Written informed consent was obtained from each study participant for repeated interviews, physical examinations, collection of biological samples, and medical record abstraction.

In total, 871 HIV-infected women and 439 uninfected women were matched on HIV risk behaviors such that ~50% of women reported injection drug use (IDU) at least once since 1985, while ~50% reported only sexual risk behavior. Women were assessed at 6-month intervals. A median of 11 visits was completed per woman, and 67% completed at least 10 visits. Women with a clinical AIDS diagnosis or any AIDS-defining opportunistic infection were not eligible for enrollment. At study entry, only 30% of HIV-infected women received monotherapy or dual therapy, and none received highly active antiretroviral therapy (HAART) [40]. The earliest available serum samples were evaluated for HCV antibodies and HCV RNA, although subsequent study visits were not routinely evaluated for HCV RNA levels. The overall prevalence of HCV antibody positivity (indicative of past or present infection) was 56.5% [41, 42].

For the current analysis, 90 antiretroviral (ART)-naïve women with HCV genotype 1 infection were included across three study groups, including 29 women with HCV mono-infection, 30 women with HIV/HCV co-infection and CD4 cell count <350 cells/mm^3^, and 31 women with HIV/HCV co-infection and CD4 cell count ≥350 cells/mm^3^. None were ever treated for HCV infection. HCV Viral RNA was extracted from 500 μL of serum using the QIAamp UltraSens Virus kit (Qiagen; Valencia, CA) and yielded ~60 μL of viral nucleic acid that was then divided into six 10 μL aliquots and frozen at -80°C until use. Additional IRB approval was obtained from the University of Cincinnati for this secondary analysis.

### NS5B amplification and next generation sequencing

RT-PCR was performed using the SuperScript III One Step RT-PCR System with the Platinum Taq DNA High Fidelity Polymerase (Invitrogen; Carlsbad, CA), 10 μL of viral nucleic acid from each sample, the forward primer 5’–ATG TCG TGT GCT GCT CAA TGT C– 3’ (corresponding to nucleotides 7588–7610 of the H77 reference strain), and the reverse primer 5’–CTA AGA GGC CGG AGT GTT TAC– 3’ (nucleotides 9386–9365). cDNA was synthesized at 50°C for 60 minutes. PCR conditions were 94°C for 3 minutes, followed by 30 cycles at 94°C for 45 seconds, 59°C for 45 seconds, and 72°C for 2 minutes, with a final elongation step at 72°C for 5 minutes. PCR products were visualized on a 1% agarose gel, and the band (~1,798 bases in length) was purified using the Gel Purification Kit (Qiagen; Valencia, CA).

Amplicon-seq was performed by the Genomics, Epigenomics and Sequencing Core at the University of Cincinnati College of Medicine. The DNA library was obtained by sonication with a Covaris S2 focused-ultrasonicator, and the sheared DNA was analyzed by Bioanalyzer DNA chip (Agilent; Santa Clara, CA). The PrepX DNA Library kit (WaferGen; Fremont, CA) and the Apollo 324 NGS automatic library prep system (WaferGen) were used for library preparation. ChIP-seq script was selected to capture all sheared fragments that were over ~80 bp, converted into blunt ends by end-repair, and adenylated at 3' ends for TA ligation to Illumina (San Diego, CA) sequencing adaptors. The ligated library was enriched by 6 cycles of PCR using index-specific primers, followed by AMPure XP bead (Beckman Coulte; Brea, CA) purification. A Bioanalyzer DNA high sensitivity chip was used to check the quality and yield of the purified library. Individually indexed libraries were proportionally pooled for clustering at a final concentration of 8 pM. Pooled libraries were clustered onto a flow cell using Illumina’s TruSeq SR Cluster kit v3 in cBot system (Illumina), followed by single read sequencing at 1x50 bp using Illumina’s TruSeq SBS kit and the Illumina HiSeq system. Sequence quality was evaluated with fastQC, and low quality reads were removed the with fastx-toolkit. Reads were then aligned with the Burrows-Wheeler Aligner allowing for up to 10 mismatches with the H77 reference sequence. Coverage and variations analyses were performed in pysamstat and alignment were visualized in IGV. Reads per patient ranged from 436,661 to 5,502,656, with an average of 2,699,629 reads per patient and an average HCV coverage of 66,464.

### Phylogenetic analysis

A consensus sequence from each individual was generated in CLC Genomics Workbench 8.5.1. Phylogenetic inference was performed using a Bayesian Markov chain Monte Carlo (MCMC) approach as implemented in the BEAST v1.10.0 program [43] under an uncorrelated log-normal relaxed molecular clock and the Hasegawa-Kishino-Yano substitution model with nucleotide site heterogeneity estimated using a gamma distribution. The MCMC analysis was run for a chain length of 100,000,000 with sampling every 10,000 generations. Results were visualized in Tracer v1.6 to confirm chain convergence, and the effective sample size (ESS) was calculated for each parameter. All ESS values were >200 indicating sufficient sampling. The maximum clade credibility tree was selected from the posterior tree distribution after a 10% burn-in using TreeAnnotator v1.8.4.

To identify phylogenetic clusters of NS5B sequences, Cluster Picker v1.2 [44] was used with bootstrap thresholds from 70% to 90% and within-cluster genetic distances from 1.5% to 4.5%. Highlighter plots were generated for each of the 3 groups through the HIV Sequence Database [45]. Signature amino acid patterns that could distinguish the three study groups from one another were evaluated with the Viral Epidemiology Signature Pattern Analysis program [46]. Codons under positive or negative selection were detected via MEME (Mixed Effects Model of Evolution) v2.0.1 as implemented in the DataMonkey program [47]. MEME is capable of identifying instances of both episodic and pervasive positive selection at the level of an individual site [48]. The Geno2phenoHCV analysis tool (http://hcv.geno2pheno.org/) and AliView program were used for mutational analysis of the sequences. A list of the drug resistance and fitness altering mutations evaluated in the current study is included in S1 Table. The NS5B consensus sequences were deposited in GenBank under the accession numbers MK903085 –MK903168.

## Results

### Patient characteristics

Of the 90 antiretroviral (ART)-naïve women selected from the parent study, the full-length NS5B could be amplified from 85 (94.4%) including 25 with HCV mono-infection, 29 with HIV/HCV co-infection and CD4 cell count <350 cells/mm^3^, and 31 women with HIV/HCV co-infection and CD4 cell count ≥350 cells/mm^3^. Age, race, and risk category were not statistically different across the three study groups (Table 1). As expected based upon the study design, the median CD4 cell counts were significantly different between the HIV/HCV co-infected women with CD4 cell count <350 cells/mm^3^ versus HIV/HCV co-infected women with CD4 cell count ≥350 cells/mm^3^ (228.0 versus 602.8 cells/mm^3^; p < 0.001). Among the HIV/HCV co-infected women, the median log_10_ plasma HIV RNA levels were higher among those in the CD4 cell count <350 cells/mm^3^ group compared to those in the CD4 cell count ≥350 cells/mm^3^ group (4.1 log_10_ copies/uL versus 3.1 log_10_ copies/uL; p < 0.001).

**Table 1:** Demographic and clinical characteristics of the 85 women from the HERS cohort with amplifiable full-length NS5B sequences based on HIV status and CD4 cell count.

	HCV mono-infected (N = 25)	HIV/HCV co-infected with CD4 <350 (N = 29)	HIV/HCV co-infected with CD4 ≥350 (N = 31)	P value
Age in years (mean, SD)	37.4 (6.3)	37.9 (5.6)	36.2 (6.1)	0.628
Race (%)				
Black	26 (96.0%)	27 (93.1%)	28 (90.3%)	0.948
White	0	1 (3.5%)	2 (6.5%)	
Hispanic	1 (4.0%)	1 (3.5%)	1 (3.2%)	
Risk cohort				
IDU	23 (92.0%)	25 (86.2%)	31 (100%)	0.088
Sexual	2 (8.0%)	4 (13.8%)	0	
CD4 cell count (median, IQR)	N/A	228.0 (161.3–277.2)	602.8 (427.8–734.4)	<0.001
Log HIV viral load (mean, SD)	N/A	4.1 (0.8)	3.1 (1.1)	<0.001
ART	N/A			
Sub-HAART		12 (41.1%)	6 (19.4%)	0.063
No ART		17 (58.6%)	25 (80.6%)	

SD–standard deviation; IQR–interquartile range; ART–antiretroviral therapy; HAART–highly active antiretroviral therapy; N/A–not applicable.

**Fig 1:**
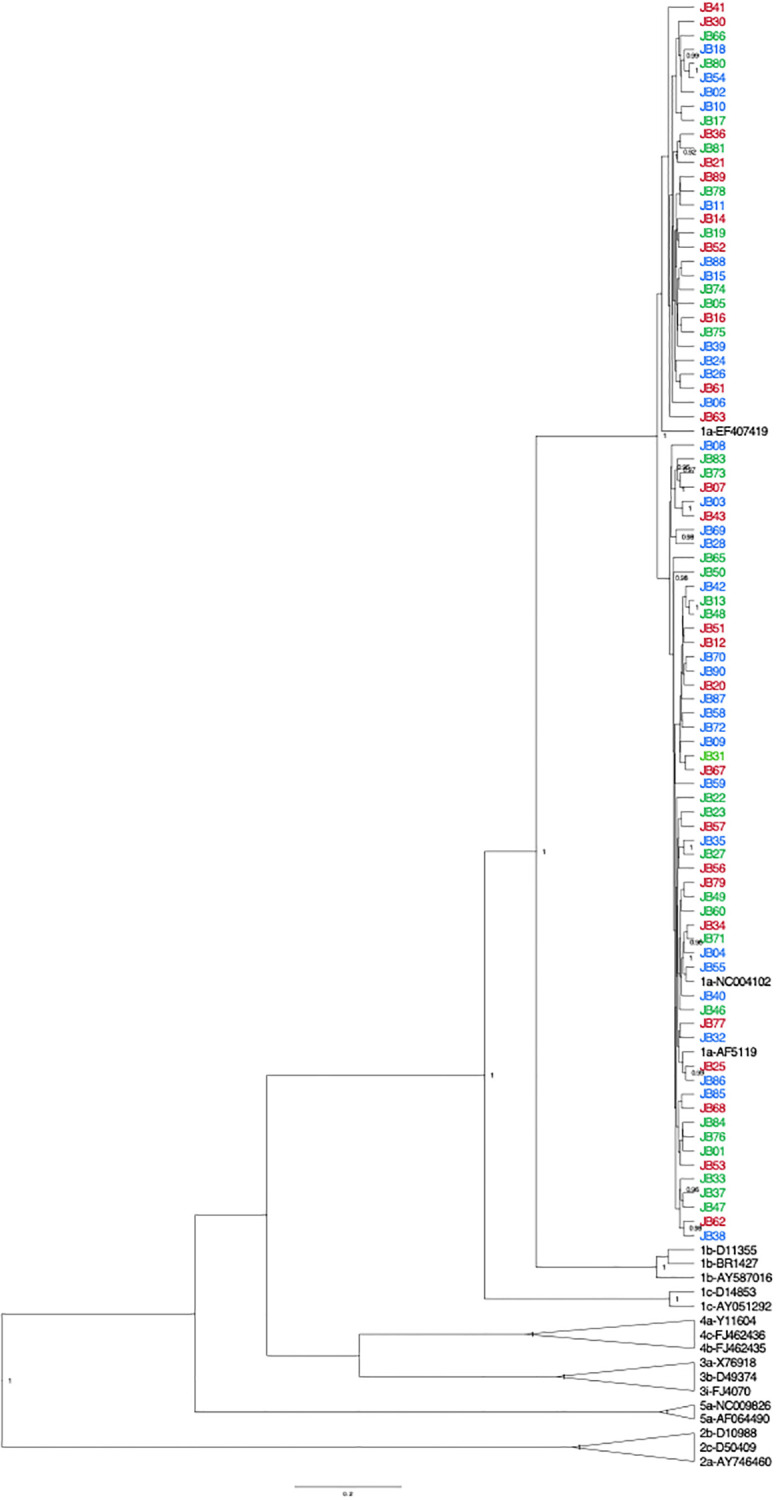
Phylogenetic analysis of full-length NS5B sequences from 85 HCV-positive women enrolled in the HERS cohort. HCV mono-infected women are shown in red. HIV/HCV co-infected women with CD4 <350 cells/mm^3^ are shown in green, while HIV/HCV co-infected women with CD4 ≥350 cells/mm^3^ are shown in blue. GenBank reference sequences are indicated by their genotype/subtype and accession number. Relevant posterior probabilities >0.90 out of 1.00 are shown. The scale bar indicates 0.02 nucleotide substitutions per site.

As shown in Fig 1, all women were infected with HCV genotype 1a. No obvious clustering of samples based on HIV co-infection or CD4 cell count was observed. Highlighter plots showed evidence of significant diversity across the entire NS5B gene in all individuals (S1 Fig). Three clusters–consisting of two NS5B sequences each–were identified. Five of these sequences were from HIV/HCV co-infected women. Cluster 1 includes JB03 (HIV/HCV co-infected with CD4 ≥350 cells/mm^3^) and JB43 (HCV mono-infected). Cluster 2 includes JB13 and JB48 (both HIV/HCV co-infected women with CD4 <350 cells/mm^3^). Cluster 3 includes JB54 (HIV/HCV co-infected with CD4 ≥350 cells/mm^3^) and JB80 (HIV/HCV co-infected with CD4 <350 cells/mm^3^).

**Fig 2:**
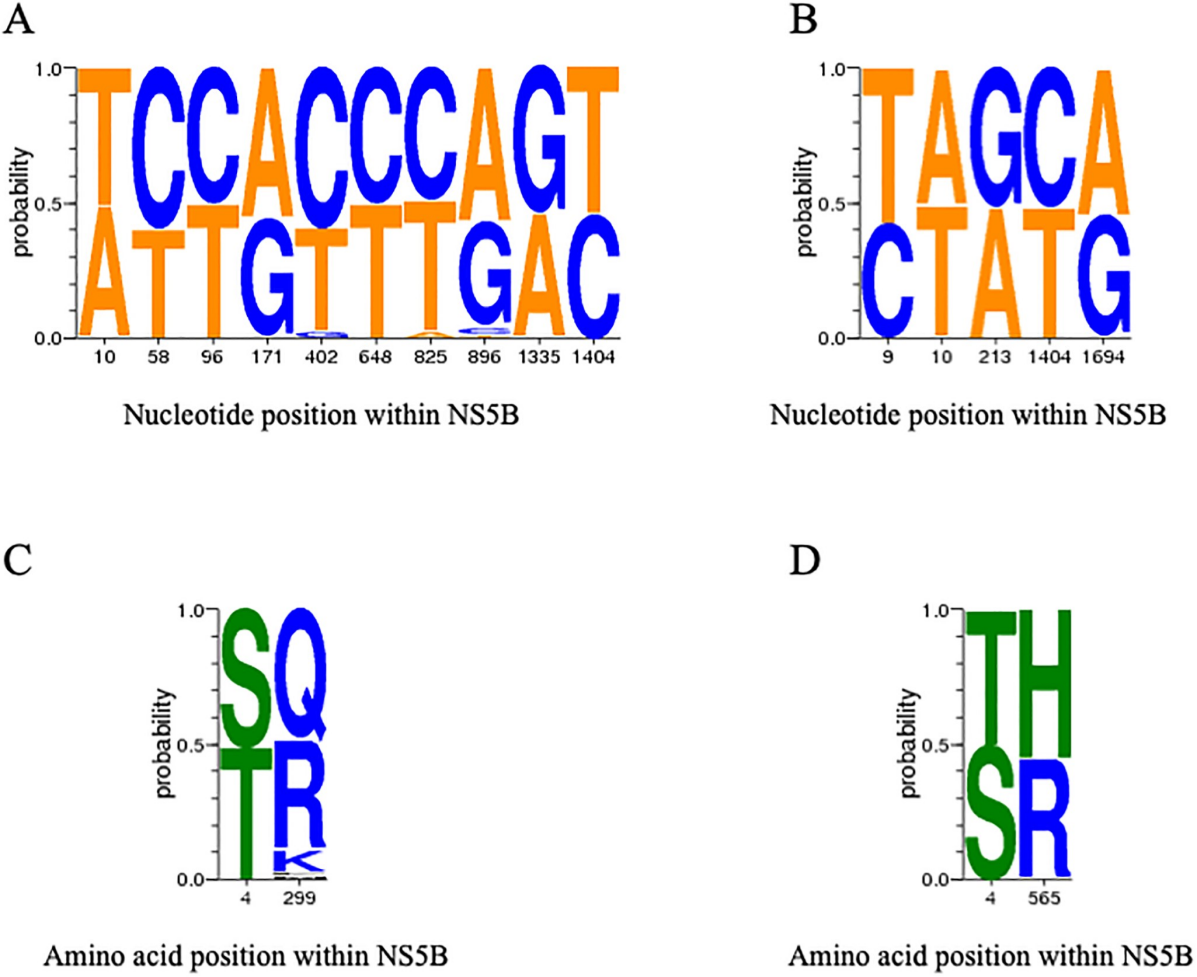
Signature pattern analysis for nucleotides (A-B) or amino acids (C-D) comparing NS5B sequences from HCV mono-infected individuals to HIV/HCV co-infected individuals regardless of CD4 cell count (A, C) or NS5B sequences from HIV/HCV co-infected individuals with CD4 cell count <350 to HIV/HCV co-infected individuals with CD4 cell count ≥350 (B, D). The height of each nucleotide or amino acid represents is relative proportion within the dataset.

Signature pattern analysis was conducted to compare NS5B nucleotide and amino acid sequences across the three study groups. When comparing HCV mono-infected individuals to HIV/HCV co-infected individuals regardless of CD4 cell count, there were 10 nucleotides (positions 10, 58, 96, 171, 402, 648, 825, 896, 1335, and 1404) and 2 amino acids (positions 4 and 299) with distinct distributions (Fig 2A and 2C). When comparing HIV/HCV co-infected individuals with CD4 <350 cells/mm^3^ to those with CD4 ≥350 cells/mm^3^, there were 5 nucleotides (positions 9, 10, 213, 1404, and 1694) and 2 amino acids (positions 4 and 565) with distinct distributions (Fig 2B and 2D).

**Fig 3:**
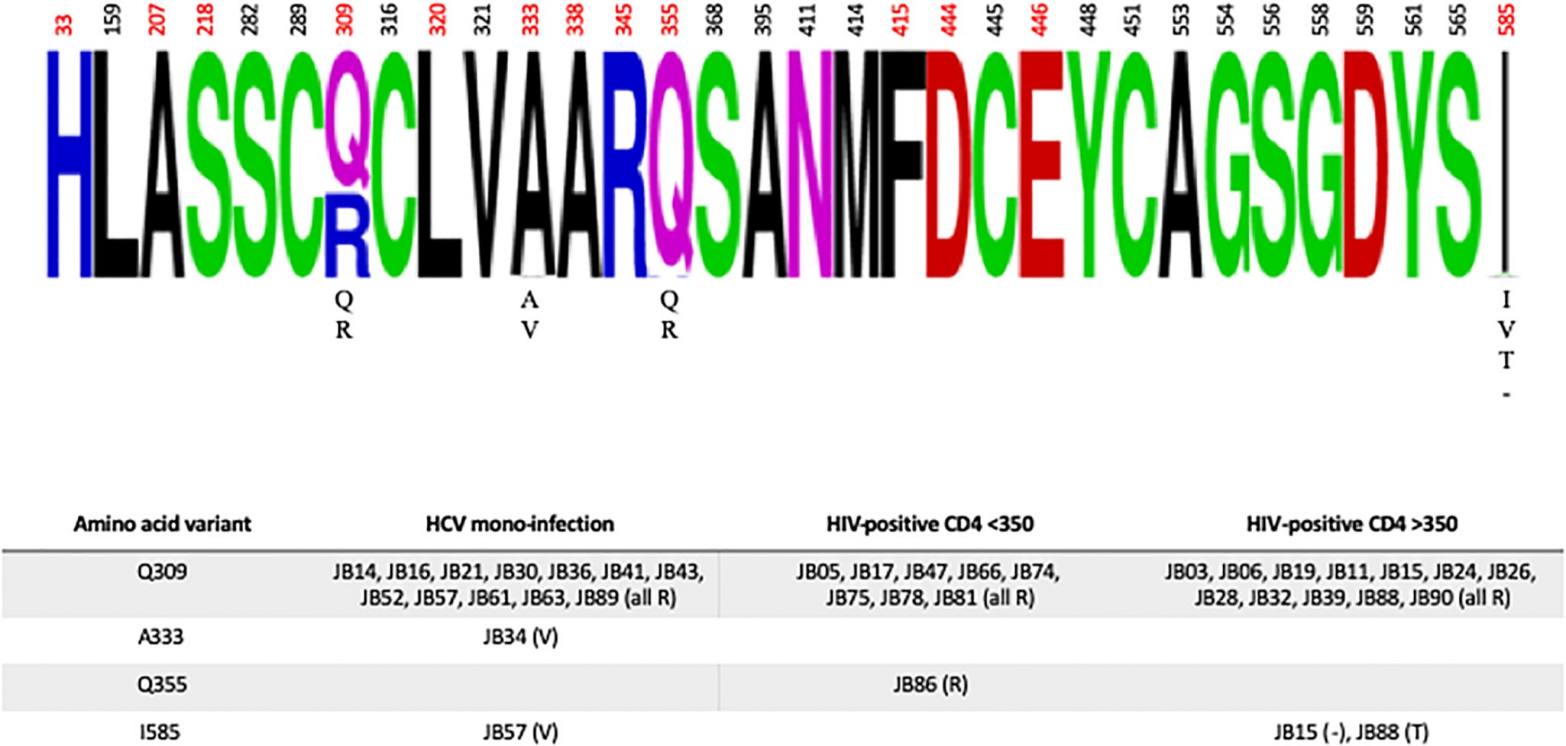
Resistance-Associated Variants (RAVs) in consensus NS5B. In the **upper panel**, relevant amino acid positions within the full-length NS5B gene are shown as a frequency plot. When present, amino acid variants at a given position are listed below the consensus. Amino acids positions in red are associated with ribavirin susceptibility and/or not evaluated by Geno2pheno. In the **lower panel**, the four amino acid positions with any variants are shown by infection group. JB numbers correspond to participant study ID. The amino acid change is noted in parentheses.

Polymorphisms at amino acid positions associated with resistance to dasabuvir and sofosbuvir were evaluated, as were sites associated with ribavirin resistance [49–52]. As shown in Fig 3, there was no variation at most sites with the exception of positions 309, 333, 355, and 585. The Q309R variant associated with ribavirin resistance was present in 12 individuals with HCV mono-infection, 8 HIV/HCV co-infected individuals with CD4 <350 cells/mm^3^, and 12 HIV/HCV co-infected individuals with CD4 ≥350 cells/mm^3^. Variation at A333 was present in 1 individual with HCV mono-infection, although the impact of this polymorphism (A333V) has not been evaluated. The Q355R variant associated with ribavirin resistance was present in 1 HIV/HCV co-infected individual with CD4 <350 cells/mm^3^. Variation at I585 was noted in 1 individual with HCV mono-infection (I585V), and 2 HIV/HCV co-infected individuals with CD4 ≥350 cells/mm^3^ (I585deletion and I585T); however, only the I585V mutation has been evaluated functionally and shown to be associated with dasabuvir resistance.

**Fig 4:**
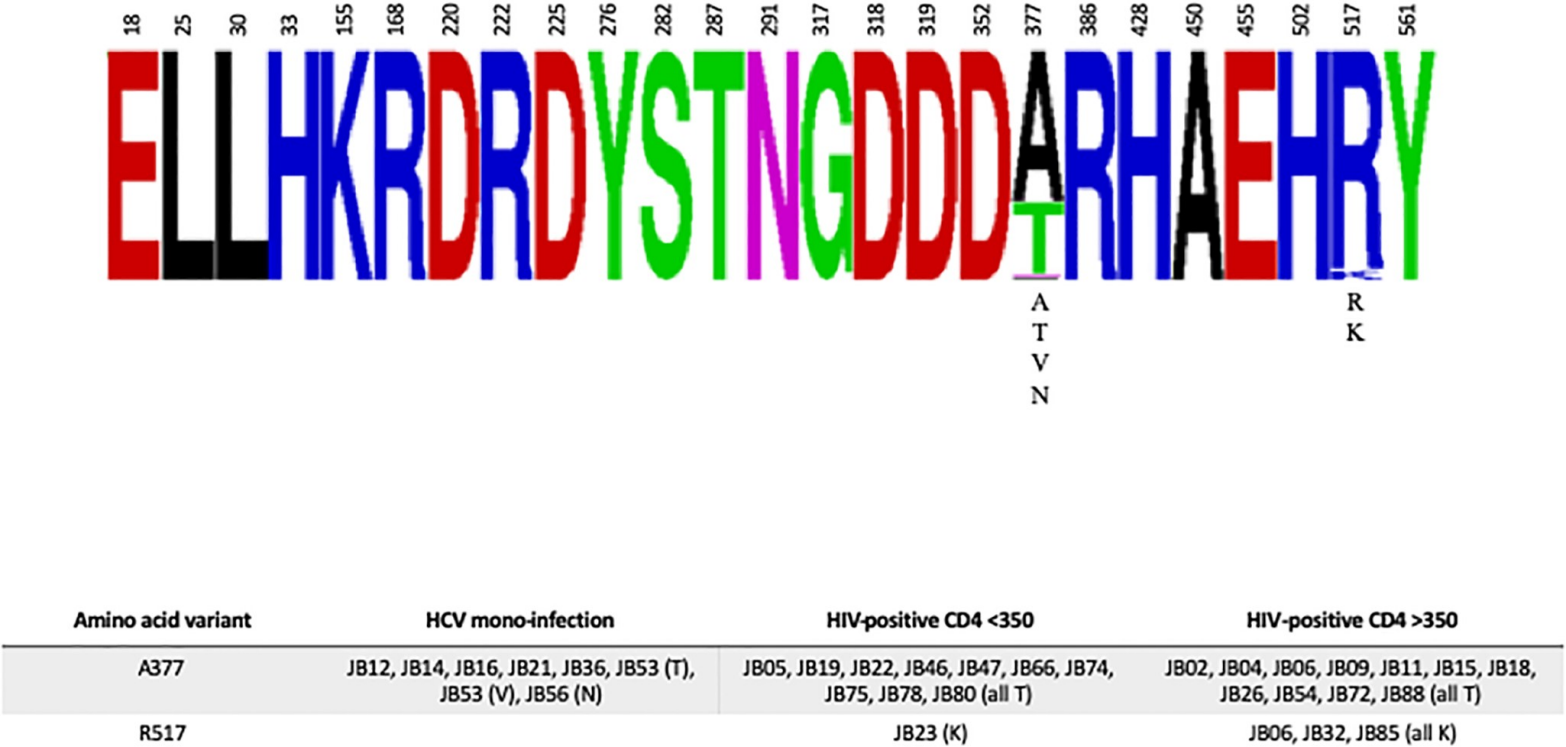
Fitness altering variants in consensus NS5B sequences. In the **upper panel**, relevant amino acid positions within the full-length NS5B gene are shown as a frequency plot. When present, amino acid variants at a given position are listed below the consensus. In the **lower panel**, the three amino acid positions with any variants are shown by infection group. JB numbers correspond to participant study ID. The amino acid change is noted in parentheses.

A number of fitness altering mutations have been reported in the literature as well. As shown in Fig 4, variation was absent at each of these positions with the exception of 377 and 517. The Q377R mutation was reported by Murayama *et al*. to increase polymerase activity in the JFH1 genotype 2 isolate [53]. However, in these genotype 1 sequences, only A, T, V, or N variants were noted. The R517K mutation was also reported by Murayama *et al*. to increase JFH1 polymerase activity. One HIV/HCV co-infected individual with CD4 <350 cells/mm^3^ and 3 HIV/HCV co-infected individuals with CD4 ≥350 cells/mm^3^ had this mutation.

**Fig 5:**
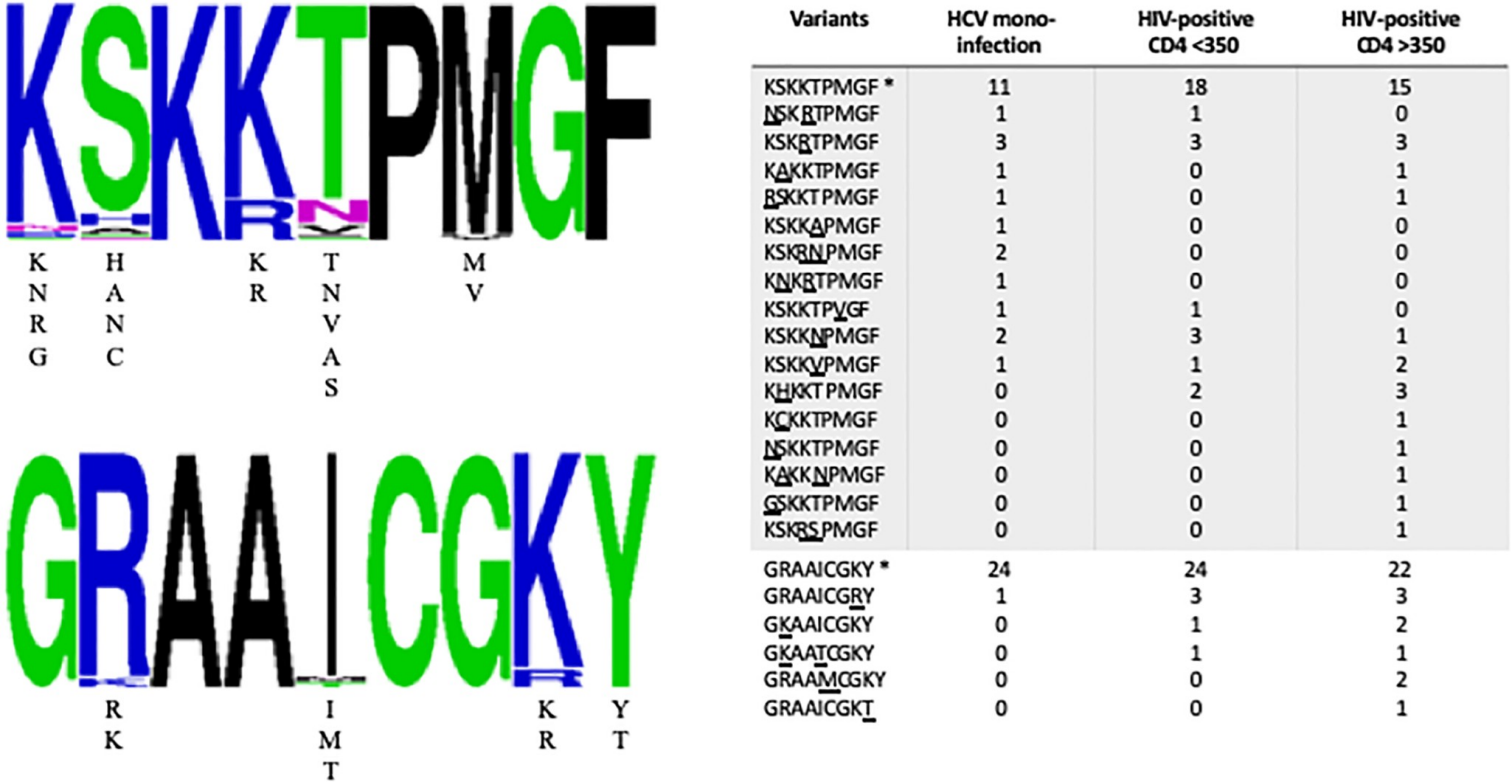
Variation in two HLA-B*57 epitopes associated with spontaneous viral clearance in consensus NS5B sequences. In the **left panel**, amino acid variants within the full-length NS5B gene are shown as a frequency plot. When present, amino acid variants at a given position are listed below the consensus. In the **right panel**, variability within these epitopes (* is wild-type; variant amino acids are underlined) is shown by infection group.

CD8^+^ T cell responses against the human leukocyte antigen (HLA) B57-restricted epitopes NS5B_2629-2637_ (KSKKTPMGF) and NS5B_2936-2944_ (GRAAICGKY) are critical for the control of HCV infection [54, 55]. As shown in Fig 5, the KSKKTPMGF was completely conserved in 44 of the 85 (51.8%) study participants. However, there were mutations in at least one position within this epitope in 14 HCV mono-infected individuals, 11 HIV/HCV co-infected individuals with CD4 <350 cells/mm^3^, and 16 HIV/HCV co-infected individuals with CD4 ≥350 cells/mm^3^. The GRAAICGKY epitope was conserved in 70 of 85 (82.4%) study participants but exhibited variability in 1, 5, and 9 individuals in the HCV mono-infected, HIV/HCV co-infected individual with CD4 <350 cells/mm^3^, and HIV/HCV co-infected individuals with CD4 ≥350 cells/mm^3^ study groups, respectively.

**Fig 6:**
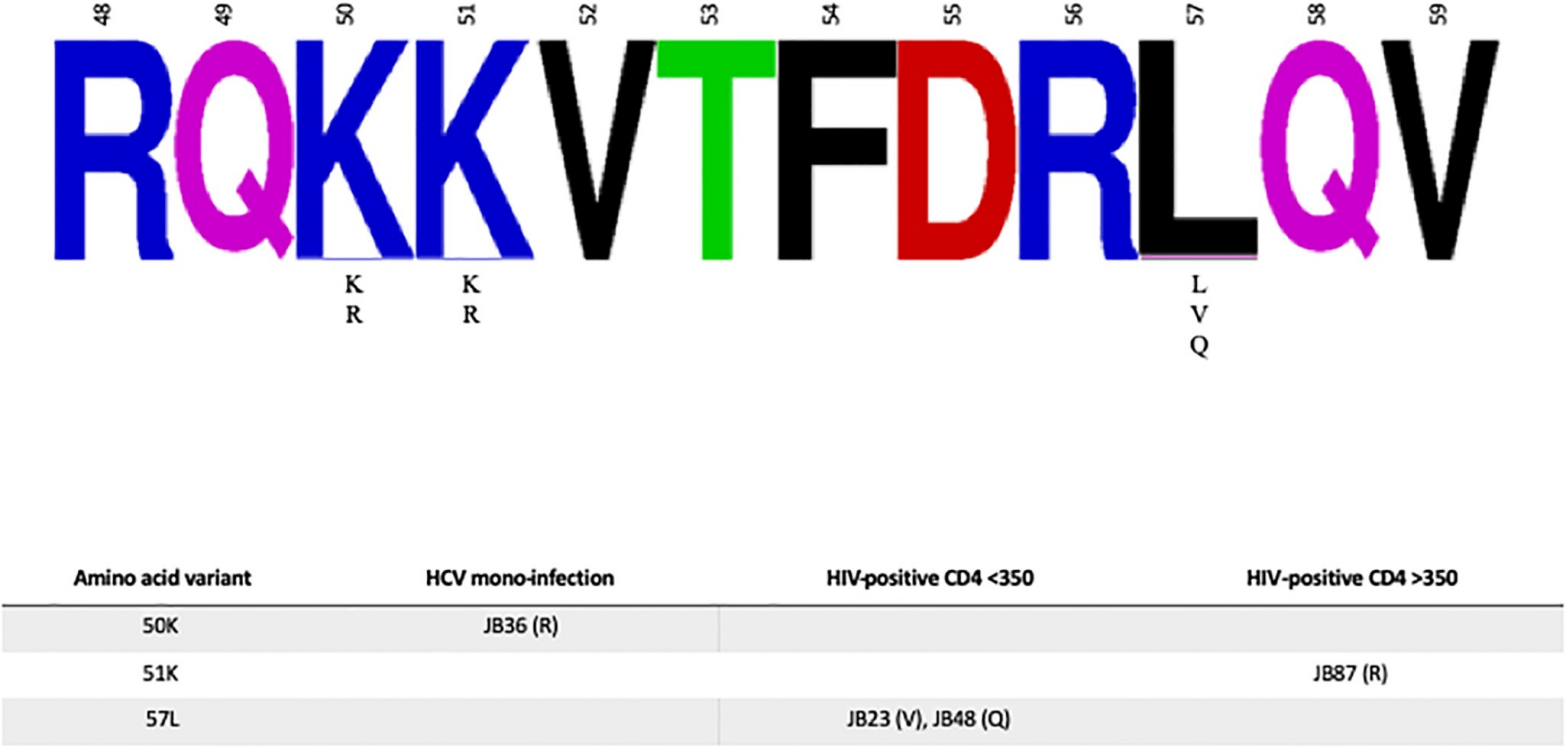
Variants within RNA channel contact points in consensus NS5B sequences. In the **upper panel**, relevant amino acid positions within the full-length NS5B gene are shown as a frequency plot. When present, amino acid variants at a given position are listed below the consensus. In the **lower panel**, the three amino acid positions with any variants are shown by infection group. JB numbers correspond to participant study ID. The amino acid change is noted in parentheses.

Vaughn *et al*. identified positions within the NS5B that contact nascent RNA during RNA synthesis [56]. As shown in Fig 6, these positions were well conserved with no variation in 81 of 85 (95.3%) study participants.

Multiple amino acid positions under positive selection pressure were detected as noted in Table 2, including 15, 21, and 15 positions in the HCV mono-infected, HIV/HCV co-infected with CD4 <350 cells/mm^3^, and HIV/HCV co-infected with CD4 ≥350 cells/mm^3^ study groups, respectively. Amino acid positions unique to a particular study group were positions 61, 177, 309, 460, and 487 in the HCV mono-infected group, positions 14, 119, 147, 197, 250, 334, and 505 in the HIV/HCV co-infected with CD4 <350 cells/mm^3^ group, and positions 10 and 542 in the HIV/HCV co-infected with CD4 ≥350 cells/mm^3^ group.

**Table 2:** Positively selected codon positions within consensus NS5B sequences based on HIV status and CD4 cell count. P values <0.10 are denoted by ✓. * Codon positions unique to the HCV mono-infected study group; ** Codon positions unique to the HIV co-infected <350 study group; *** Codon positions unique to the HIV co-infected ≥ 350 study group.

Codon position	HCV mono-infected	HIV/HCV co-infected with CD4 <350	HIV/HCV co-infected with CD4 ≥350
10 ***			✓
14 **		✓	
46	✓	✓	
61 *	✓		
89	✓	✓	✓
97	✓	✓	✓
116	✓	✓	✓
119 **		✓	
134		✓	✓
147 **		✓	
177 *	✓		
179	✓	✓	✓
188		✓	✓
197 **		✓	
205		✓	✓
209	✓	✓	✓
212	✓	✓	✓
250 **		✓	
308		✓	✓
309 *	✓		
326	✓	✓	✓
334 **		✓	
376	✓	✓	✓
460 *	✓		
487 *	✓		
505 **		✓	
542 ***			✓
543	✓	✓	✓

## Discussion

Extensive interpatient diversity is a defining characteristic of HCV infection. While structural regions of the viral genome have been studied extensively, similar analyses of non-structural regions are limited. Nonetheless, non-structural genomic regions such as NS5B are responsible for RNA replication, as well as critical virus-virus and virus-cell interactions. Non-structural regions are also subject to selection via the immune system and/or antiviral therapies. In this well characterized cohort, we previously observed a higher median genetic distance for the HIV/HCV co-infected women compared to the HCV mono-infected women. Immune selection pressure was positively correlated with CD4 cell count but negatively correlated with HCV RNA levels [29]. In another study of viral diversity, we observed that genetic distances were higher for E1/HVR1 compared to NS5B in both the sera and peripheral blood mononuclear cells (PBMCs) [57]. Evidence of possible viral compartmentalization in the PBMCs was observed suggesting that viral adaptation to a unique extracellular microenvironment(s) may be required for efficient replication and viral persistence.

There were several noteworthy findings in the current analysis. First, diversity was observed across the entire NS5B gene in all individuals. This may seem counterintuitive given the essential role played by the RNA-dependent RNA polymerase in the HCV life cycle; however, structural and functional constraints on this genomic region are not absolute and many nucleotide/amino acid positions–even those within the NS5B gene–exhibit some variability. Second, there was no obvious clustering of NS5B sequences by HIV status or CD4 cell count. While HIV co-infection and immune function are known to impact HCV RNA levels and HCV diversity [58–63], such influences are not sufficiently strong to generate highly adapted viruses that circulate in defined clusters in a population-based study such as that conducted here. Third, the frequencies of several nucleotides and amino acids where different in the three study groups, suggesting that the selection pressures acting upon these clinical groups may be different as well; however, this requires confirmation in other cohorts and at-risk populations. Fourth, mutations associated with commonly used NS5B inhibitors such as dasabuvir and sofosbuvir were rare. This is not unexpected given the recent development of these direct-acting antivirals compared to the sample collection period (1993 to 2000). Fifth, despite a number of fitness altering mutations being reported *in vitro*, the presence of such mutations *in vivo* was uncommon. Sixth, immune epitopes associated with spontaneous viral clearance exhibit some polymorphism, although the functional consequences of these changes were not evaluated in this study.

Several limitations of the current study should be noted. First, the study population focused on genotype 1a as the most common HCV genotype found within the United States, and these findings may not be applicable to other genotypes. Second, the HERS cohort included only women; therefore, certain comparisons may not be generalizable to men; however, there are no compelling data to suggest that HCV diversity differs appreciably by biological sex. Third, the HERS cohort was not specifically designed to address liver disease, and liver biopsies were not routinely performed. However, we previously evaluated FIB-4 scores–a non-invasive index of liver fibrosis–in the HERS cohort [64]. Fourth, covariation of the NS5B sequence with other viral proteins is known to occur [16, 17, 20, 65], although we have not evaluated other genomic regions in this cohort extensively. Nonetheless, mapping these covariant positions on to available protein crystal structures would provide additional insight into which positions may come in direct contact with positions within the same protein or positions within other viral proteins such as NS3/4A and NS5A that are requisite components of the HCV replication complex [65, 66]. Finally, NGS was utilized to derive high quality sequence data but consensus NS5B sequences were evaluated in the current study; thus, minor drug resistance variants and the presence or absence of multiple NS5B variants within a single individual should be evaluated in subsequent studies.

## Supporting information

S1 Table List of the drug resistance and fitness altering mutations evaluated in the current study. (XLSX)

S1 Fig Highlighter plots for full-length NS5B sequences from 85 HCV-positive women enrolled in the HERS cohort. The prototype HCV isolate H77 is included as the reference to which all study sequences are compared.
(TIFF)
