# Supplementary figures and images for: Diversity of the hepatitis C virus NS5B gene during HIV co-infection
Source: PLoS One. 2020 Aug 4;15(8):e0237162. doi: 10.1371/journal.pone.0237162 (PMC7402467; doi:10.1371/journal.pone.0237162)

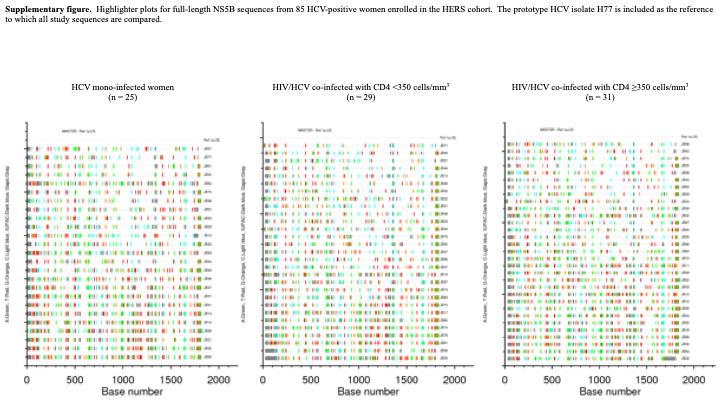

Supplement: S1 Fig — The prototype HCV isolate H77 is included as the reference to which all study sequences are compared. (TIFF) [file pone.0237162.s002.tiff]
